# Comparative Study on Assisted Solvent Extraction Techniques for the Extraction of Biologically Active Compounds from *Sideritis raeseri* and *Sideritis scardica*

**DOI:** 10.3390/molecules28104207

**Published:** 2023-05-20

**Authors:** Marika Mróz, Edyta Malinowska-Pańczyk, Agnieszka Bartoszek, Barbara Kusznierewicz

**Affiliations:** Department of Food Chemistry, Technology and Biotechnology, Faculty of Chemistry, Gdańsk University of Technology, 11/12 Narutowicza St., 80-233 Gdańsk, Poland; marika.mroz@pg.edu.pl (M.M.); edyta.malinowska-panczyk@pg.gda.pl (E.M.-P.)

**Keywords:** *Sideritis raeseri*, *Sideritis scardica*, extraction, UPLC-HRMS, antioxidants, MAE, USAE, HPE

## Abstract

The plants in the *Sideritis* genus are postulated to exhibit several important medicinal properties due to their unique chemical composition. To isolate the targeted phytochemical compounds, the selection of a suitable extraction method is of primary importance. In this work, a comparative study on the phytochemical profiles of various *Sideritis raeseri* and *Sideritis scardica* extracts has been carried out. An untargeted metabolomics approach based on ultra-high performance liquid chromatography coupled with high-resolution mass spectrometry was applied to investigate the metabolic differences between extracts obtained by conventional extraction and extractions assisted by microwaves, ultrasounds and high pressure. Additionally, the influence of extraction solvents on HPLC antioxidant profiles obtained following the derivatization of analytes with ABTS reagent was evaluated. A total of 102 metabolites have been putatively identified. The major secondary metabolites groups were classified as flavonoids, terpenoids, phenylethanoid glycosides and phenolic acids. The main antioxidants in the extracts were isoscutellarein and hypolaetin derivatives as well as verbascoside and chlorogenic acid. The results showed that 70% ethanol was the most effective extractant for different classes of phytochemicals including antioxidants. In addition, extraction supported with microwaves, ultrasounds or high pressure improved the overall recovery of metabolites by about 3 times compared to the conventional extraction method.

## 1. Introduction

The Mediterranean region, the Balkan Peninsula and the Middle East possess suitable climate conditions for house plant communities rich in herbs and shrubs from the *Lamiaceae* family, including the *Sideritis* genus [1]. In traditional medicine, infusions or decoctions prepared with the flowering aerial parts of the *Sideritis* species were widely used for the treatment of the common cold, cough, gastrointestinal disorders and for the healing of wounds [2,3]. *Sideritis scardica*, endemic to the Balkan Peninsula [4], is known also as ironwort. Depending on the region of origin, in Bulgaria, infusions from this herb are known as “Mursalski tea”, “Pirinski tea” or “Alibotushki tea”. In the Republic of North Macedonia, it is commonly named “Sharplaninsi chaj”, whereas in Greece, it can be called “Greek Mountain tea” or “Greek Olympus Tea” [5,6,7]. *Sideritis raeseri* is another popular herb variety endemic to the Balkans and the Iberian Peninsula, also cultivated in Greece [8,9]. All these mentioned plants contain a variety of health-promoting phytochemical constituents, including phenolic acids, flavonoids, phenylethanoid glycosides and terpenoids [1]. Previous studies indicated miscellaneous biological properties of the *Sideritis* species: anti-inflammatory, gastroprotective, cytotoxic, antimicrobial and antioxidant [7,10]. These chemopreventive properties can be fully exploited in the pharmaceutical or food industry only with the condition that effective isolation of the bioactive phytochemicals is ensured.

In the case of a complex matrix such as herbs, which often contain bioactive substances of very different polarities, the extraction solvent should be carefully selected. When extracting specific compounds, the solvent should match the polarity of the target compound. The simultaneous recovery of multiple compounds requires a more versatile solvent. Generally, in the food industry, non-toxic and easy-to-use solvents are preferred for plant extraction. Water is the safest and cheapest green solvent; nonetheless, its application is limited to polar compounds. In the case of less polar molecules, a higher efficiency can be achieved with the aid of organic solvents. The most satisfactory results are usually obtained with binary solvent mixtures (e.g., water and an organic solvent), which also have been shown to be more efficient and environmentally friendly than pure organic solvents [11]. In order to improve the effectiveness of the bioactive substance extraction, aqueous/organic solvents must be combined at the appropriate ratio. For example, despite the low solubility of polyphenols in water, its addition to an organic solvent increases the diffusivity of the solvent within the matrix [12,13]. Flavonoid glycosides are more water-soluble than aglycones, but these two chemical forms can be extracted simultaneously with a mixture of water and alcohol or pure alcohols. It has been observed that less polar flavonoids, such as flavanones, flavanols, isoflavones and methylated flavones are more soluble in ethyl acetate, diethyl ether, chloroform and dichloromethane. However, due to the toxicity of these solvents, they must be handled with caution and need to be evaporated to a safe limit permissible for food [11].

Conventional solvent extractions such as Soxhlet extraction and maceration exhibit some fundamental disadvantages, being time-consuming, costly, and not ecological. These drawbacks have directed research towards more cost-effective and greener methods for the extraction of bioactive compounds from plant material. The proposed alternative techniques include the following: ultrasound-assisted extraction, microwave-assisted extraction, pressurized liquid extraction and pressurized hot water extraction. These extraction techniques oftentimes employ elevated temperature, which on one hand increases solubility and mass transfer due to the reduced viscosity of the solvent used, but on the other hand may be destructive for thermosensitive compounds. Another alternative green extraction technique is supercritical fluid extraction, typically with carbon dioxide, a solvent generally recognized as safe (GRAS). This type of extraction is conducted at low temperatures (<5 °C); however, the slow diffusion of the solute from the solid matrix makes this process time-consuming [14]. The remedy may be high-pressure extraction conducted in sub-zero temperatures, which on top of speeding up the process prevents the thermal degradation and loss of bioactivity of the extracted compounds. The advantages of using high-pressure extraction for the isolation of polyphenols, including flavonoids, have been noticed previously [15]. It was observed that a high-pressure extraction time of less than 20 min gave a similar recovery of solutes as 2 h boiling or 3 h supercritical CO_2_ extraction. Moreover, the mentioned alternative extraction methods are recognized as environmentally friendly.

This study was aimed at finding out the method of preparation of *Sideritis* extracts that would ensure the maximum recovery of health-beneficial compounds from these plants. The aerial parts of *Sideritis raeseri* and *Sideritis scardica* are traditionally brewed with hot water for the preparation of infusions. In the presented study, it was hypothesized that the addition of an appropriate amount of a non-toxic organic solvent such as ethanol to the water during extraction may improve the extraction efficiency of the desired phytochemicals. The untargeted metabolomics approach based on ultra-high performance liquid chromatography coupled with high-resolution mass spectrometry (UHPLC-HRMS) fingerprinting was applied to investigate the differences in the composition of metabolites isolated from *S. raeseri* and *S. scardica* using extractants of different polarities achieved by changing the proportions of water and ethanol. Additionally, the influence of extraction solvents on HPLC antioxidant profiles obtained by the derivatization of analytes with an ABTS radical was evaluated. The composition of the phytochemicals in the *Sideritis* genus was the subject of several publications [7,10]. However, the number of detected and monitored metabolites was generally limited to the main groups of *Sideritis* metabolites such as phenolics, flavonoids and phenylethanoid glycosides, which resulted in the identification of only about twenty or thirty metabolites [3,4,16,17,18,19]. The analytical approach presented here enabled the tracking of over 100 metabolites belonging to different classes of phytochemicals. In addition, for the first time, according to our best knowledge, antioxidant profiling was used for species from the *Sideritis* genus, which made it possible to identify the most important phytochemicals responsible for the antioxidant properties of these plants. Another research goal was to determine whether and to what extent the use of assisted extraction with microwaves, ultrasounds or high pressure would affect the extraction efficiency and profile of phytochemicals. Former studies on extraction methods used for the isolation of *Sideritis* metabolites usually were limited to determinations of the total flavonoid content, the total phenolic content, the total antioxidant activity of extracts and selected individual compounds [1,2,19,20,21,22]. In this study, consideration has been extended to the assessment of the impact of the extraction method on the detailed phytochemical composition in *S. raeseri* and *S. scardica* extracts.

## 2. Results and Discussion

### 2.1. Phytochemical Composition of Sideritis scardica and Sideritis raeseri

The phytochemical composition of the extracts from Sideritis scardica and Sideritis raeseri was determined by HR-LC-ESI-Orbitrap-MS analysis in negative ion mode. The negative ion mode was selected for final data processing due to the lower background noise and matrix interferences, more fragment ion patterns and a larger number of identifiable phytochemicals. During the MS/MS experiments, a “data dependent scan” mode was used in which MS software selects the precursor ions corresponding to the most intense peaks in the LC-MS spectrum. Some of the main peaks were tentatively attributed according to the accurate masses, characteristic fragmentation patterns and retention times in comparison with literature data on the Sideritis genus. Finally, 102 phytochemicals were successfully identified, including 31 flavonoids, 14 phenolic acids and 14 terpenoids—mostly iridoid glycosides, 13 phenylethanoid glycosides and other compounds such as sugar acids and saccharides, carboxylic acids, etc. Relevant information on all of the compounds identified is provided in Table 1.

Flavonoids and their derivatives are the dominant phytochemicals in the *Sideritis* species [18]. In this study, about one-third of identified compounds were classified as flavonoids. These compounds were mostly glycosides and acetyl glycosides of flavonoids and their methylated forms, which is characteristic of the *Sideritis* species [29]. The main flavonoid aglycones found in studied plants were hypolaetin (*m*/*z* 301), methylhypolaetin (*m*/*z* 315), isoscutellarein (*m*/*z* 285), methylisoscutellarein (*m*/*z* 299) and apigenin (*m*/*z* 269). The characteristic fragmentation pattern of the acetylated flavonoid glycosides is the loss of the acetyl residue (*m*/*z* 42), H_2_O (*m*/*z* 18) and hexose (*m*/*z* 162) units. The abundant [M−H−342]^−^ ions indicate the presence of two hexose units. Diacetylated derivatives were characterized by another neutral loss of acetyl residue. Compounds **49**, **61** and **78** with [M−H]^−^ at *m*/*z* 625.14099, 667.15155 and 709.16180 have been characterized as hypolaetin allosyl glycoside, hypolaetin acetyl allosyl glucoside and hypolaetin diacetyl allosyl glucoside. The common fragment ion of *m*/*z* 301.035 represented deprotonated hypolaetin [27]. Methylhypolaetin glycosides shared the characteristic 315.051 fragment, which was observed in compounds: **60**, **66** and **83** ([M−H]^−^ at *m*/*z* 639.15674, 477.10394, 723.17773) and two isomers of methylhypolaetin acetyl glucoside—**72** and **77** ([M−H]^−^ at *m*/*z* 681.16711 and 681.16766). Compounds **56**, **63**, **69** and **82** showed pseudo-molecular ions [M−H]^−^ at *m*/*z* 609.14581, 447.09323, 651.15662 and 693.16705, respectively. They all gave a common fragment ion of *m*/*z* 285.04025 which could be attributed to isoscutellarein (**81**, [M−H]^−^at *m*/*z* 285.04037). Compounds **70**, **76**, **80** and **88** with [M−H]^−^ at *m*/*z* 665.17224, 623.16193, 665.17242 and 707.18292 produced characteristic fragments of *m*/*z* 299.056 that corresponded to the methylated form of isoscutellarein. Fragments of *m*/*z* 269.04568 originating from deprotonated apigenin were registered in compounds **41**, **53**, **67**, **68**, **86** and **92** that gave pseudo-molecular ions [M−H]^−^ at *m*/*z* 593.15137, 593.15155, 431.09845, 635.16150, 577.13519 and 283.06125, respectively. Compound **86** also gave characteristic fragments of *m*/*z* 145.02838 which correspond to a loss of apigenin and hexose unit [M−H−270−162]^−^ and 413.0878 was produced by the loss of coumaroyl moiety [M−H−164]^−^, hence it was identified as apigenin-7-O-(6″-O-4-coumaroyl)-beta-glucoside (echinacin). Additionally, this compound was previously reported in *S. scardica* and *S. raeseri* [23,24].

Another abundant class of compounds found in *Sideritis* were phenylethanoid glycosides [18]. An investigation of the fragmentation pattern allowed for the identification of the phenylethanoid glycosides group by their common loss of masses: 162 Da, 146 Da, 18 Da, 15 Da and 179 Da, corresponding to hexose, rhamnose, H_2_O, Me and caffeoyl units, respectively. Among all detected compounds, 13 were classified as phenylethanoid glycosides. Compounds **52** and **55** with precursor ions at *m*/*z* 623.19812 shared the typical fragmentation pattern for verbascoside and isoverbascoside with 461.16638/461.16678 *m*/*z* fragments [M−H−162]^−^ arising from the loss of caffeoyl moiety, which also was observed as a separate 179 *m*/*z* ion. The fragment ion of *m*/*z* 315.10938 represented the further loss of deoxyhexose and the 135.04388 fragment resulted from the subsequent loss of hexose and water. Compound **45** with [M−H]^−^ at *m*/*z* 639.19348 was identified as β-hydroxyverbascoside, as it showed a similar fragmentation pattern to verbascoside, with the additional characteristic ion at *m*/*z* 151.03902. Compound **27** ([M−H]^−^ at *m*/*z* 461.16660) was identified as a decaffeoyl-verbascoside, also known as verbasoside [30]. Compound **58** with a precursor ion [M−H]^−^ at 799.26605 showed the fragmentation pattern of a glycosidic derivative of a methylether of verbascoside with 637.21533 and 623.21814 fragments due to the loss of hexose unit and subsequent loss of methyl group. The proposed molecular formula (C_14_H_20_O_7_) for compound **30** ([M−H]^−^ at *m*/*z* 299.11365) with an error of −0.25 ppm and its fragmentation pattern was consistent with these of salidroside. Compound **59**, identified as allysonoside, showed a precursor ion at *m*/*z* 769.25604, the fragment ion observed at *m*/*z* 593.20935 corresponded to the loss of the feruloyl unit [M−H]−176]^−^, whereas the *m*/*z* 461.16599 fragment was produced by the subsequent loss of apiosyl unit [M−H−176−132]^−^. Other fragments commonly observed for allysonoside [25,28], except the fragment of *m*/*z* at 637.2131, did not occur under applied analytical conditions. Compound **48** gave a precursor ion [M−H]^−^ at *m*/*z* 785.25116 which corresponded to the chemical formula C_35_H_45_O_20_^−^ that could be attributed to the deprotonated form of echinacoside [28]. Compound **51** yielded the base peak at *m*/*z* 755.74200 which was accurate for either samioside or lavandulifolioside. The formed product ions at *m*/*z* 593.20941 and 623.19867 occur in both compounds, so fragmentation did not allow for distinguishing them. Compound **64** ([M−H]^−^ at *m*/*z* 637.21381), with the characteristic fragment of *m*/*z* at 461.1666 [28], was tentatively identified as leucoseptoside A. Compound **75** ([M−H]^−^ at *m*/*z* 651.22961) showed fragments produced by the loss of feruloyl unit [M–H−176]^−^ and was identified as martynoside as it is one of the main phenolic glycosides of the *Sideritis* species [10,19].

In the case of phenolic acids, the major one was chlorogenic acid (compound **37)** with the main characteristic ions originating from caffeic and quinic acid fragments (179 and 191 Da). Compound **20** ([M−H]^−^ *m*/*z* at 169.01329) was identified as gallic acid as it gave a characteristic fragment of *m*/*z* 125.02325. Compound **21,** with a parent ion at *m*/*z* 183.02913, was identified as methyl gallate. Compound **28** ([M−H]^−^ *m*/*z* at 153.01819) was identified as gentistic acid, as its fragmentation pattern corresponded to that reported previously [26]. Compound **42,** with base peaks at *m*/*z* 179.03415, was tentatively identified as caffeic acid. Compounds **33** and **34** ([M−H]^−^ *m*/*z* at 315.07239 and 299.07736) were assigned as gentistic acid glucoside and salicylic acid glucoside, based on fragments produced by the loss of a hexose [M−162−H]^−^.

The loss of glucose [M−162]^−^ and characteristic loss of 182 amu indicate the presence of iridoid glycosides. Compounds **19** and **22** with pseudo-molecular ions at *m*/*z* 361.11432 and 523.16693 were identified as monomelittoside and melittoside, respectively. Fragments of *m*/*z* 163.03896 corresponding to a deprotonated coumaric acid ion led to the identification of compound **39** ([M−H]^−^ *m*/*z* at 669.20361) as 10-*O*-(E)-*p*-coumaroylmelittoside. Compound **43** was tentatively assigned as ajugoside due to the presence of the precursor ion at 389.14542 *m*/*z* and its prior presence in some other *Sideritis* species [3,18]. Compounds **29** and **44** with precursor ions at *m*/*z* 375.12970 and 417.14059 were identified as 8-epiloganic acid and 7-*O*-acetyl-8-epiloganic acid. Compound **23,** with the pseudo-molecular ion [M−H]^−^ at *m*/*z* 373.11420, generated fragments of *m*/*z* 123.04385, 149.05965 and 89.02296, which was consistent with the fragmentation path of geniposidic acid. Among registered substance peaks, several belonged to abietane diterpenoids, which produced characteristic fragment ions due to the loss of carbon dioxide (44 Da), carbon monoxide (−28 Da), water (−18 Da) and methyl radical (15 Da). Compounds **89** ([M−H]^−^ *m*/*z* at 345.17105), **94** ([M−H]^−^ *m*/*z* at 329.17609) and **97** ([M−H]^−^ *m*/*z* at 331.20456) were identified as rosmanol, carnosol and carnosic acid, respectively.

### 2.2. Comparative Analyses of Phytochemical Variation between Extracts with Different Polarities from Sideritis raeseri and Sideritis scardica

**Figure 1:**
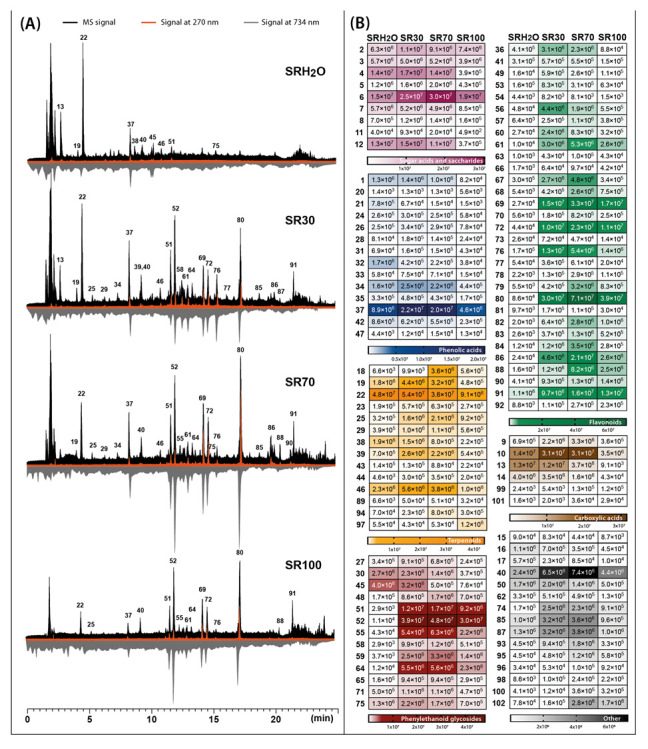
Total ion chromatograms obtained by LC-Q-Orbitrap in negative mode (black) combined with chromatograms registered by UV-Vis detector at 270 nm (orange) and antioxidant profiles registered at 734 nm after post-column derivatization with ABTS (grey) (**A**), assembled with heat maps representing the mean MS peak area value of the identified compounds in four different *Sideritis raeseri* extracts: SRH_2_O—water extract; SR30—30% ethanol extract; SR70—70% ethanol extract; SR100—ethanol extract (**B**). For identity of peaks, see Table 1.

**Figure 2:**
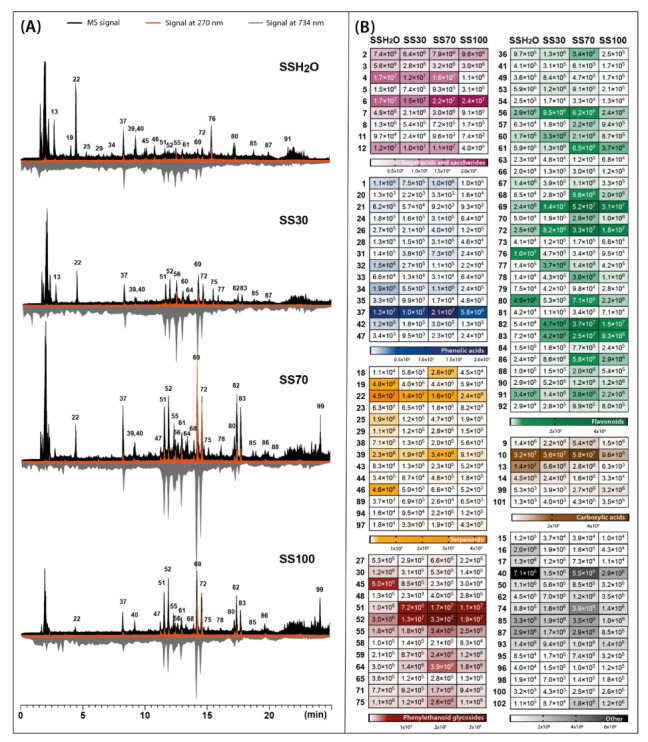
Total ion chromatograms obtained by LC-Q-Orbitrap in negative mode (black) combined with chromatograms registered by UV-Vis detector at 270 nm (orange) and antioxidant profiles registered at 734 nm after post-column derivatization with ABTS (grey) (**A**), assembled with heat maps representing the mean MS peak area value of the identified compounds in four different *Sideritis scardica* extracts: SSH_2_O—water extract; SS30—30% ethanol extract; SS70—70% ethanol extract; SS100—ethanol extract (**B**). For identity of peaks, see Table 1.

Four parallel extractions were carried out for two species of *Sideritis* studied with extractants of different polarities, regulated by changes in the proportions of water and ethanol. These solvents were chosen as non-toxic and easy-to-handle for plant extraction preferred in the food and pharmaceutical industry. The phytochemical profiles of each extract accompanied by the corresponding heat map with the signal intensity of individual phytochemicals detected in four different *S. raeseri* and *S. scardica* extracts are presented in Figure 1 and Figure 2, respectively.

**Figure 3:**
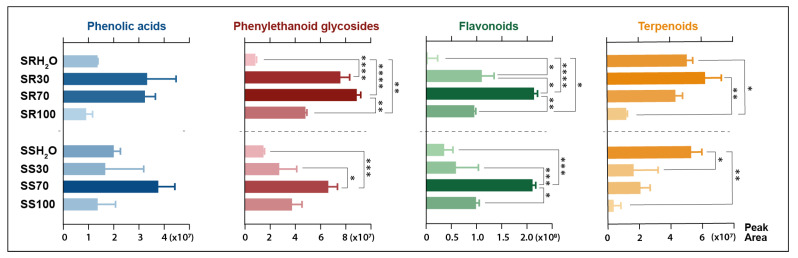
Extraction efficiency of major classes of bioactive phytochemicals from two *Sideritis* species with four different solvents based on sum of peak areas of compounds assigned to the appropriate classes. SS—*Sideritis scardica*; SR—*Sideritis raeseri*; H_2_O—water extract; 30—30% ethanol extract, 70—70% ethanol extract; 100—ethanol extract. Asterisks representing p-value classification refer to: (*) *p* ≤ 0.05; (**) *p* ≤ 0.01; (***) *p* ≤ 0.001; (****) *p* ≤ 0.0001.

The main phytochemicals detected in two *Sideritis* species were phenolic compounds such as phenylethanoid glycosides, flavonoid glycosides and phenolic acids. In the case of the class of phenylethanoid glycosides and phenolic acids, verbascoside (compound **52**) and chlorogenic acid (compound **31**) were predominant compounds. The class of flavonoids was mainly represented in *S. raeseri* by 4′-*O*-methylisoscutellarein 7-*O*-[6‴-*O*-acetyl]-allosyl(1→2)glucoside (compound **80**) and in *S. scardica* by isoscutellarein 7-*O*-[6‴-acetyl]-allosyl(1→2)-glucoside (compound **69**). Another important class of phytochemicals present in the *Sideritis* extracts studied were terpenoids represented mostly by melittoside (compound **22**) belonging to the iridoid glycosides. Considering the recovery of compounds belonging to these main classes, calculated as the sum of peak areas retrieved from MS analysis, four different solvents were compared (Figure 3).

The highest total content of phenylethanoid glycosides was achieved with the use of 30% or 70% ethanol aqueous solution as a solvent. The best extractant for flavonoids was without a doubt 70% ethanol, as the total flavonoid area was twice greater than with the use of 30% ethanol or pure ethanol. The extraction of flavonoids with water was the least effective. The best solvent in the case of phenolic acids could not be clearly defined due to the lack of statistical differences between the tested samples. The optimal ethanol concentration for the high-rate extraction of total phenols from *S. raeseri* was established as 70% in a previous study [1]. Moreover, when targeting phenolic compounds from *S. raeseri*, it was proven that aqueous ethanolic extract could be further enriched by successive extraction with ethyl acetate [2]. In previous research reports, total phenolic content was significantly higher in *S. raeseri*, whereas total flavonoid content was higher in *S. scardica* [18]. In this study, the approximate content of flavonoids and phenolics based on the total peak area did not differ between these varieties. Terpenoid compounds showed the greatest affinity for water. The peak area of terpenoids from *S. raeseri* was on a similar level for all water-containing extracts. In the case of *S. scardica*, the extraction of terpenoids with water was significantly better than with any other studied extractant. Terpenoids are a wide class of compounds; in the studied plants, they were predominantly classified as iridoids, mostly in the form of glycosides, which explains the stronger affinity for polar solvents. The high total area of iridoid compounds suggested their presence in both studied varieties, although they were not reported previously in *S. scardica* [18]. Other representatives of this class found in studied extracts were abietane diterpenoids such as carnosol and carnosic acid. They are common in various plants from the *Lamiaceae* family, but in the *Sideritis* species, their concentration is rather low. Due to their non-polar nature, these compounds’ abietane diterpenoids tended to be better extracted with ethanolic solvents. Higher levels of these compounds observed in aqueous-ethanolic extracts of *S. raeseri* compared to *S. scardica* may be explained by the difference between the terpenoid profiles in these two varieties.

**Figure 4:**
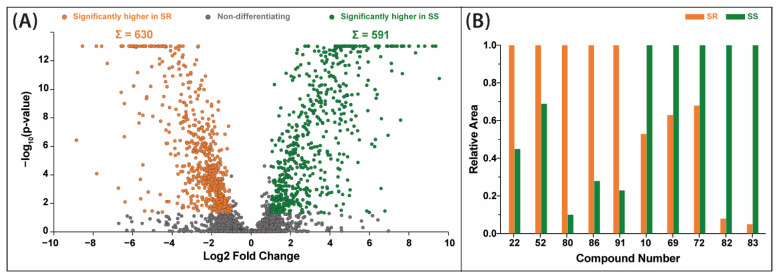
Volcano plot combining the results of fold change (FC) analysis and t-tests comparing *Sideritis raeseri* (SR) and *Sideritis scardica* (SS) extracts prepared with 70% ethanol (**A**) juxtaposed with a bar graph showing the relative peak area of the 10 characteristic metabolites differentiating the SS and SR varieties (the contents of each higher one converted to 1.0) (**B**). For identity of peaks, see Table 1.

Differential analysis of *Sideritis scardica* and *Sideritis raeseri* extracts performed by a fold change analysis coupled with a *t*-test indicated that the largest number of differentiating compounds was present in 70% ethanolic extracts (Figure 4A). For these extracts, a total of 630 substance peaks were assigned at significantly higher levels in the *S. raeseri* variety, estimated based on the MS peak areas. Conversely, 591 substance peaks were assigned in favor of the *S. scardica* variety. Among them, 10 main compounds which can be the basis for distinction between these varieties were distinguished (Figure 4B). In the case of *S. raeseri,* the area of peaks attributed to 4′-*O*-methylisoscutellarein 7-*O*-[6‴-*O*-acetyl]-allosyl(1→2)-glucoside (**80**), verbascoside/isoverbascoside (**52**), melittoside (**22**), apigenin-7-*O*-(6″-*O*-4-coumaroyl)-glucoside (**86**) and (−)-usnic acid/eupatorin (**91**) were significantly greater compared to *S. scardica.* In contrast, the other five compounds’ areas were notably greater in *S. scardica*. These compounds are the following: isoscutellarein 7-*O*-[6‴-*O*-acetyl]-allosyl(1→2)-glucoside (**69**), quinic acid (**10**), isoscutellarein 7-*O*-[6‴-*O*-acetyl]-allosyl(1→2)-[6″-*O*-acetyl]-glucoside (**82**), 4′-*O*-methylhypolaetin 7-*O*-[6‴-*O*-acetyl]-allosyl(1→2)-glucoside (**72**) and 4′-*O*-methylhypolaetin 7-*O*-[6‴-*O*-acetyl]-allosyl-(1→2)[6′′-*O*-acetyl]-glucoside (**83**).

The presence of compounds exhibiting antioxidant activity in the plant material has become an important aspect in defining its health-promoting qualities. In the presented study, the antioxidant profiles were generated for *S. raeseri* and *S. scardica* extracts prepared with extractants of different polarities. Post-column addition of the ABTS reagent during HPLC analysis of the extracts causes the reduction of blue-green colored radicals by the analytes leaving the column, which is recorded at 734 nm as chromatograms with characteristic negative peaks (Figure 1A and Figure 2A—grey chromatograms). The obtained results indicated that antioxidant profiles also depended on the solvent used for extraction. In the case of 70% ethanolic extracts, the signals corresponding to antioxidants were the most intensive, while water extracts showed the least intensive signals. The dominant antioxidants in *S. scardica* were compounds **69** and **72,** identified as isoscutellarein 7-*O*-[6‴-*O*-acetyl]-allosyl(1→2)-glucoside and 4′-*O*-methylhypolaetin 7-*O*-[6‴-*O*-acetyl]-allosyl(1→2)-glucoside (Figure 2A). These compounds also contributed to the antioxidant activity of *S. raeseri*, but not as strongly as compounds **80** (4′-*O*-methylisoscutellarein 7-*O*-[6‴-*O*-acetyl]-allosyl(1→2)glucoside) and **52** (verbascoside) (Figure 1A). According to Krgović et al. [2], the antioxidant activity of *S. raeseri* was the most positively correlated with the content of 4′-*O*-methyl-isoscutellarein 7-*O*-[6‴-*O*-acetyl-*β*-D-allopyranosyl-(1→2)]-*β*-D-glucopyranoside and 4′-*O*-methyl-hypolaetin 7-*O*-[6‴-*O*-acetyl-*β*-D-allopyranosyl(1→2)]-6‴-*O*-acetyl-*β*-D-glucopyranoside, identified also in our study as compounds **80** and **83**, respectively. However, in our study, the antioxidant activity of compound **83** was not observed at all in *S. raeseri* extracts, whereas in *S. scardica* extracts, it was relatively weak compared to other antioxidants. In both varieties, the antioxidant activity of the chlorogenic acid assigned as compound **37** was also noticeable. Another important metabolite that showed relatively strong antioxidant activity was phenylethanoid glycoside, assigned as compound **51,** and a peak originating from compound **61** (hypolaetin 7-*O*-[6‴-*O*-acetyl]-allosyl(1→2)-glucoside). The antioxidant activity of chlorogenic acid, phenylethanoid glycosides and flavonoids derived from isoscutellarein and hypolaetin have also been reported in other studies [10,31,32,33].

The significant influence that solvents with different polarities have on yield, composition profile and antioxidant activity has been clearly shown in this study for the extracts from *S. raeseri* and *S. scardica*. The obtained results on metabolite identification and antioxidant profiling indicated that 70% ethanol aqueous solution turned out to be the most effective extractant for the bioactives present in the studied *Sideritis* species. However, the aerial parts of *S. scardica* and *S. raeseri* are commonly used to prepare infusions (known as mountain tea). According to Irakli et al. [34], to prepare an *S. scardica* infusion with the highest level of phenolics, flavonoids and antioxidant activity, the temperature of water should be between 87.5 and 99.8 °C and the contact time of the dried plant material with water should be 10 min. However, the obtained results clearly indicate that the addition of ethanol to water significantly increases the extraction efficiency of various groups of bioactive phytochemicals. The water-ethanol solution is recognized as a low-toxicity and environmentally friendly extraction medium, therefore, as a GRAS (Generally Recognized as Safe) system, it can be used in the food, cosmetics and pharmaceutical industries. Therefore, a 70% aqueous ethanol solution was selected for further research aimed at increasing the efficiency of analytes’ solubilization by using assisted extraction methods.

### 2.3. Comparative Analyses of Phytochemical Variation between Extracts from Conventional and Assisted Solvent Extractions

For environmental, economic and safety reasons, there is a need to develop alternative, more ecological methods of extraction, enabling sustainable and selective recovery of valuable compounds and overcoming the limitations of conventional methods. For this reason, in the next stage of our research, the comparative analysis of phytochemical variability between extracts from conventional (CSE) and assisted solvent extractions such as microwave-assisted extraction (MAE), ultrasound-assisted extraction (USAE) and high-pressure extraction (HPE) was carried out using LC-Q-Orbitrap HRMS.

**Figure 5:**
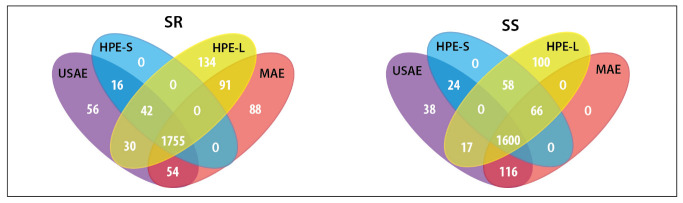
Venn diagrams showing the number and relations between the significantly better-extracted metabolites, in terms of peaks areas obtained by using alternative solvent extraction (ASE) compared to the extracts obtained with conventional solvent extraction method. SR—*Sideritis raeseri*; SS—*Sideritis scardica*; CSE—conventional solvent extraction; USAE—ultrasound-assisted extraction; MAE—microwave-assisted extraction; HPE—high-pressure extraction (S—20 min of extraction; L—18 h of extraction).

**Figure 6:**
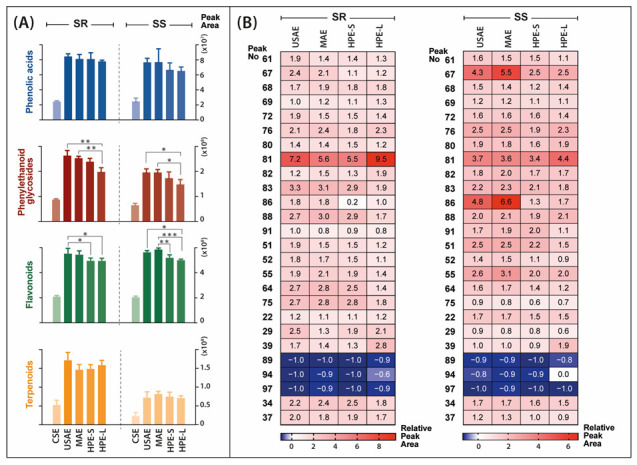
Comparison of the extraction methods in terms of bioactive compound recovery. Bar graphs represent total peak areas of compounds isolated with different extraction methods and grouped into classes characteristic for *Sideritis* species (**A**). The heatmaps show the relative peak areas of main identified bioactive metabolites obtained by different extraction methods. The results are presented in relation to the conventional solvent extraction areas, which have been assumed to equal 0.0 (**B**). Red indicates an improvement in bioactive extraction, blue means deterioration. The abbreviations refer to: SR—*Sideritis raeseri*; SS—*Sideritis scardica*; CSE—conventional solvent extraction; USAE—ultrasound-assisted extraction; MAE—microwave-assisted extraction; HPE—high-pressure extraction (S—20 min of extraction; L—18 h of extraction). Asterisks representing *p*-value classification are as follows: (*) *p* ≤ 0.05; (**) *p* ≤ 0.01; (***) *p* ≤ 0.001. For identity of peaks, see Table 1.

In this step, all extractions were conducted with the 70% aqueous ethanol solution. The MS data acquired from the analysis of extracts obtained by different methods were imported into Compound Discoverer 2.1 software for the identification of extracted metabolites. At this stage, a total of 3639 substance peaks were detected in both *Sideritis* varieties studied. Each extraction was conducted in triplicate, which allowed for the performance of a statistical analysis. For differential analysis purposes, each alternative solvent extraction (ASE) method was compared to conventional solvent extraction (CSE) by combined fold change analysis and t-tests, taking into account all of the substance peaks. Venn diagrams show the number and relationship of substance signals significantly differentiating the USAE, MAE, HPE-S and HPE-L extracts from CE extracts (Figure 5). Regardless of the alternative extraction method used, the significant increase of areas attributable to 1600 and 1755 individual metabolites was observed in *S. scardica* and *S. raeseri*, respectively. Thus, all of the alternative methods had a significant impact on the complexity of the extracts. Additionally, the impact of the extraction method on the recovery of the main groups of bioactive compounds characteristic of the *Sideritis* species and individual bioactives was investigated (Figure 6A,B). All of the alternative extraction methods studied have improved the overall recovery of metabolites compared to the conventional extraction method. The improvement was especially noticeable in the case of the extraction of phenylethanoid glycosides and flavonoids, which were the main bioactives in the *Sideritis* species. Considering these two classes, the total areas obtained with USAE and MAE were significantly greater than while using both types of HPE. In the case of MAE, the yields of phenolics and flavonoids may decrease as a result of prolonged exposure to high temperature [20,35]. Hence, the importance of introducing a cooling phase between each irradiation step when it is not possible to control the temperature of the process should be recognized. On the other hand, according to Šavikin et al. [1], the high temperature used during USAE had a positive influence on the total phenolics content in *S. raeseri* extracts, with an optimum level at 62.75 °C. No significant differences occurred in the total areas of phenolic acids and terpenoids. Moreover, the extraction of some terpenoid compounds, including rosmanol (**89**), carnosol (**94**) and carnosic acid (**97**), did not benefit from any of the studied alternative solvent extraction methods. As shown in Figure 6B, the relative peak areas determined for these compounds were lower than 0, which means that in this case, ASE was worse than CSE. Considering all ASE methods, HPE-S did not introduce any new substance signals (blue fields in Figure 5). However, prolonging the high-pressure extraction time to 18 h (HPE-L) resulted in the extracts with the greatest number of additional substances differentiating from CSE (134 in SR extracts and 100 in SS extracts). However, this may be a result of the presence of low-molecular-weight degradation products. However, considering individual compounds, longer exposure to high pressure led to higher recovery of scutellarein or its isomer isoscutellarein (**81**) compared to other extraction methods. In this study, the HPE turned out to be the least effective of the ASE methods. This may be due to the used parameters of the process. The temperature was −20 °C, while oftentimes the HPE is conducted at room temperature or higher (even up to 60 °C). Additionally, the pressure (193 MPa) was relatively low. As previously reported [36], the pressure in HPE can reach up to 600 MPa or even 1000 MPa [37].

## 3. Materials and Methods

### 3.1. Plant Material

The flowering aerial parts of *Sideritis raeseri* and *Sideritis scardica* used for this study were obtained from ecological producer (GRECO Bio Products, Thessaloniki, Greece). According to manufacturer’s information, the plant material was traditionally air-dried without exposure to sunlight.

### 3.2. Determination of Moisture Content

The moisture content was determined with the aid of a moisture analyzer RADWAG MAX 50/1 (Radom, Poland).

### 3.3. Preparation of Extracts

Before extraction, dried herbs were finely ground in a laboratory mill (2 min) and mixed thoroughly before weighing. Moisture content of the plant material was 5.46 and 5.12% in *S. raeseri* and *S. scardica*, respectively. Each extraction was performed with accurately weighed 5 g of plant material suspended in 110 mL of solvent (1:22, *w*:*v*).

#### 3.3.1. Conventional Solvent Extraction (CSE)

For CSE, plant material was mixed with four extractants of different polarities, adjusted with varying proportions of water and ethanol. The solvents for extraction were water, ethanol, and 30% and 70% *v/v* aqueous ethanol solutions. Extractions were carried out at room temperature, except for the aqueous extract, which was prepared with boiling water to resemble typical infusions. The contact time of the plant material with the solvent was 10 min. After extraction, the samples were centrifuged at 13,000 rpm for 5 min and supernatants were collected. The extraction was conducted in triplicate with each extraction solvent.

#### 3.3.2. Assisted Solvent Extractions (ASE)

For each assisted extraction, the plant material was mixed with 70% ethanol aqueous solution. For microwave-assisted extraction (MAE), the method described by Alipieva et al. [20] with slight modifications was used. The microwave extraction was performed in a Bartscher 610.835 microwave oven (Salzkotten, Germany) with a power of 360 W. To avoid super-boiling, the extraction process was carried out for a total of 2 min, with alternating 10 s of microwave irradiation and 10 s cooling periods. The ultrasound-assisted extraction (USAE) was performed in POLSONIC SONIC-3 ultrasonic bath (Warsaw, Poland) with an ultrasonic frequency of 40 kHz and power of 310 W. The samples were treated with ultrasound for 20 min at 25 °C. For high-pressure extraction (HPE), the samples were suspended in 70% ethanol aqueous solution and transferred to sterile plastic, flexible containers, which were deaerated and sealed. The samples were pressurized at 193 MPa and −20 °C. Compression step lasted 90 min and samples were kept in these conditions for either 20 min (HPE-S) or 18 h (HPE-L). Decompression was performed for 30 min. The extraction was carried out in high-pressure equipment designed at the Department of Food Chemistry, Technology and Biotechnology, Gdansk University of Technology, and built by DS-Technology Ltd. (Slupsk, Poland). The details of the procedure have been previously described by Malinowska-Pańczyk et al. [38]. All extractions were conducted in triplicate. After extraction, the samples were centrifuged at 13,000 rpm for 5 min and the supernatants were collected.

### 3.4. LC-Q-Orbitrap HRMS Analysis

Extracts of *S. scardica* and *S. raeseri* were analyzed by the UltiMate 3000 UPLC system (Thermo Scientific Dionex) consisting of a quaternary pump, well plate autosampler, column compartment equipped with Kinetex^®^ column (150 × 4.6 mm, 5 μm, Phenomenex) and PDA detector, coupled with a high-resolution Thermo Q-Exactive^TM^ Focus quadrupole-Orbitrap mass spectrometer (Thermo, Bremen, Germany). Chromatographic system was controlled with Chromeleon 7.2.8 software (Thermo Fisher Scientific, Waltham, MA, USA). Mobile phases used for elution were as follows: A—water acidified with formic acid (0.1%) and B—acetonitrile acidified with formic acid (0.1%). The flow rate of 0.8 mL/min was used in all separations. The gradient started with 5% B and then increased to 40% B within 18 min, then reached 100% B in 20 min and was kept at this level up to 25 min. The column was conditioned with the initial mobile phase for 7 min period and the system was flushed with injection of MeOH:H_2_O (1:1, *v*:*v*) after each analysis. The injection volume was 2 μL. Ionization of the analytes in negative ion mode was performed with HESI. The flow rate of sheath gas, auxiliary gas and sweep gas was set at 35 arb, 15 arb and 3 arb, respectively. The spray voltage was 2.5 kV, and S-lens RF level was 50. Capillary temperature and heater temperature were 350 °C and 300 °C, respectively. The mass range for the full MS scan was 120–1200 *m*/*z* with resolution of 70,000 FWHM, and AGC target at 2 × 10^5^ and max inject time of 100 ms. MS^2^ parameters were as follows: 17,500 FWHM (resolution), 3 *m*/*z* (isolation window, 30 eV (collision energy), 2 × 10^5^ (AGC target) and 100 ms (max inject time). Data processing was done using Compound Discoverer 2.1 software and Freestyle 1.3 software.

### 3.5. Antioxidant Profiling by Post-Column Derivatization with ABTS

To obtain profiles of antioxidants present in *S. scardica* and *S. raeseri* extracts, HPLC-PAD system (1200 series, Agilent Technologies, Wilmington, DE, USA) coupled with Pinnacle PCX Derivatization Instrument (Pickering Laboratories Inc., Mountain View, CA, USA) was used. The detailed method has been described previously by Kusznierewicz et al. [39]. The chromatographic column and conditions of chromatographic separation were the same as in the case of LC-HRMS analysis. However, in this case, the eluate leaving the PAD detector was mixed with methanolic ABTS solution stream (1 mM, 0.1 mL/min) and directed to the reaction loop of derivatization instrument (1 mL, 130 °C). Then, the eluate stream was led further to the UV-Vis detector (Agilent Technologies, Wilmington, DE, USA) where reduction of ABTS radical by extract components was monitored at 734 nm.

### 3.6. Statistical Analysis

Microsoft Excel and GraphPad Prism 8 software were used for statistical analysis. Two-way analysis of variance (ANOVA) and Šídák’s multiple comparisons test were carried out while comparing the MS peak areas of individual classes of compounds between different extracts. All differences with *p* < 0.05 were considered statistically significant. Differential analysis of metabolites was performed with Compound Discoverer 2.1 software. Differential metabolites were defined as metabolites with log_2_fold change ≥ 1. In order to separate differential metabolites from not-significantly differential metabolites, a threshold of −log_10_(*p*) < 0.05 was used.

## 4. Conclusions

The overall aim of this study was to thoroughly investigate the total phytochemical profile of *S. scardica* and *S. raeseri*. The UHPLC-HRMS analysis led to the identification of 102 metabolites, which is twice as many as reported so far in the literature. Besides aqueous extracts similar to the traditional *Sideritis* infusions, also known as mountain tea, herein three other extracts prepared with non-toxic solvents had been studied. The most complex and rich in phytochemicals were the extracts obtained with the binary solvent, i.e., 70% ethanol aqueous solution. Additionally, the antioxidant profiles of aqueous-ethanolic extracts indicated the advantage of binary solvent extraction over single solvent extraction in terms of health-beneficial compound recovery. In addition, three different assisted solvent extraction techniques were used in an attempt to further improve the extraction of *Sideritis* metabolites. The same extractant for all methods was selected based on previous results. Methods used included well-established techniques in phytochemical recovery such as ultrasound-assisted extraction, microwave-assisted extraction and less commonly used high-pressure extraction. The proposed assisted solvent extraction methods for *Sideritis* gave promising results as the recovery of the metabolites was three times higher in comparison to conventional solvent extraction. The obtained results indicate the legitimacy of further research that will enable the industrial application of such *Sideritis* extracts.

## Figures and Tables

**Table 1:** Major compounds identified by LC-Q-Orbitrap HRMS in different extracts of Sideritis raeseri and Sideritis scardica.

No.	Compound	RT [min]	Formula	Theoretical [M−H]^−^	Experimental [M−H]^−^	Δmass [ppm]	MS/MS	Class
**1**	* Cinnamaldehyde	1.87	C_9_H_8_O	131.04969	131.04895	5.6	72.0076; 58.0284; 70.0284; 71.0236; 71.0124; 113.034	PA
**2**	* *O*-Hexosyl-hexose	1.92	C_12_H_22_O_11_	341.10839	341.10884	−1.3	89.0229; 59.0124; 101.0229; 179.0552; 71.0124; 119.0336	S
**3**	* Glucose	1.95	C_6_H_12_O_6_	179.05557	179.05515	2.3	59.0124; 71.0124; 72.9916; 58.0046; 87.0072; 55.0174	S
**4**	* Gluconic acid [4]	1.97	C_6_H_12_O_7_	195.05048	195.05016	1.7	75.0073; 59.0124; 72.9916; 71.0124; 87.0073; 105.0179	S
**5**	* Raffinose	1.97	C_18_H_32_O_16_	503.16122	503.16127	−0.1	179.0550; 89.0229; 503.1614; 221.0659; 101.0229; 161.0443	S
**6**	* D-(+)-Maltose	2.01	C_12_H_22_O_11_	341.10839	341.10873	−1.0	59.0124; 89.0229; 71.0124; 101.0230; 113.0229; 119.0334	S
**7**	* Xylonic acid	2.01	C_5_H_10_O_6_	165.03992	165.03940	3.1	75.0073; 59.0124; 72.9916; 71.0124; 87.0073; 76.0106	S
**8**	* L-Threonic acid	2.03	C_4_H_8_O_5_	135.02935	135.02867	5.0	75.0073; 71.0124; 72.9917; 59.0124; 55.017; 58.0046	S
**9**	* Quinic acid + coniferin	2.05	C_19_H_34_O_17_	533.17178	533.17200	−0.4	191.0552; 192.0585; 85.0279; 59.0124; 191.3149; 93.0328	CA
**10**	D-(−)-Quinic acid [4,23]	2.07	C_7_H_12_O_6_	191.05557	191.05522	1.8	85.0280; 93.0331; 87.0073; 59.0124; 109.0282; 81.0331	CA
**11**	* L-(+)-Tartaric acid	2.16	C_4_H_6_O_6_	149.00862	149.00821	2.7	75.0074; 72.9917; 59.0125; 69.0331; 71.0125; 66.0084	S
**12**	* DL-Malic acid	2.32	C_4_H_6_O_5_	133.01370	133.01413	−3.2	71.0124; 72.9917; 59.0124; 72.0158; 72.9958; 115.0022	S
**13**	Citric acid [4]	2.83	C_6_H_8_O_7_	191.01918	191.01900	1.0	87.0074; 111.0075; 85.0281; 57.0332; 67.0175; 59.0125	CA
**14**	* Oxaloglutarate	2.88	C_7_H_8_O_7_	203.01918	203.01895	1.1	71.0124; 79.0174; 69.0331; 97.0281; 95.0124; 72.9916	CA
**15**	* L-Tyrosine	2.90	C_9_H_11_NO_3_	180.06607	180.06589	1.0	152.917; 167.9038; 72.0076; 119.0491; 122.9579; 93.0333	AA
**16**	* Uridine	2.92	C_9_H_12_N_2_O_6_	243.06171	243.06221	−2.0	82.0285; 110.0235; 66.0335; 168.0146; 122.0234; 118.9650	N
**17**	* Guanosine	3.65	C_10_H_13_N_5_O_5_	282.08385	282.08356	1.0	150.0411; 133.0144; 126.0297; 108.0190; 107.0350; 151.0457	N
**18**	* Melittoside isomer	4.21	C_21_H_32_O_15_	523.16630	523.16688	−1.1	89.0229; 119.0336; 179.0551; 59.0124; 71.0124; 113.0231	I
**19**	* Monomelittoside [17]	4.23	C_15_H_22_O_10_	361.11348	361.11332	0.4	59.0124; 89.0229; 71.0124; 99.0074; 101.0229; 155.0339	I
**20**	* Gallic acid	4.44	C_7_H_6_O_5_	169.01370	169.01329	2.4	69.0331; 125.0232; 124.0152; 97.0281; 79.0174; 95.0123	PA
**21**	Methyl gallate [24]	4.55	C_8_H_8_O_5_	183.02935	183.02913	1.2	137.0233; 136.0155; 108.0204; 109.0284; 124.0154; 111.0075	PA
**22**	Melittoside [18,25]	4.65	C_21_H_32_O_15_	523.16630	523.16693	−1.2	89.0229; 59.0124; 119.0337; 71.0124; 101.0230; 113.0231	I
**23**	* Geniposidic acid	4.87	C_16_H_22_O_10_	373.11348	373.11420	−1.9	123.0438; 149.0596; 89.0229; 59.0124; 71.0124; 121.0646	I
**24**	* Glucovanillyl alcohol	5.06	C_14_H_20_O_8_	315.10800	315.10784	0.5	153.0545; 112.9842; 138.0313; 68.9944; 71.0124; 154.0585	PA
**25**	* Methylscutelloside	5.47	C_16_H_26_O_11_	393.13969	393.14047	−2.0	127.0388; 167.0705; 149.0595; 89.0229; 121.0646; 59.0123	I
**26**	* Vannilic acid glucoside [18]	5.69	C_14_H_18_O_9_	329.08726	329.08786	−1.8	108.0203; 152.0104; 167.0339; 123.0438; 153.0137; 109.0237	PA
**27**	* Decaffeoylverbascoside	6.32	C_20_H_30_O_12_	461.16591	461.16660	−1.5	113.0231; 112.9842; 135.0439; 89.0229; 71.0124; 68.9943	PEG
**28**	* Gentisic acid [26]	6.47	C_7_H_6_O_4_	153.01879	153.01819	3.9	108.0203; 109.0283; 68.9943; 91.0174; 58.9900; 110.0314	PA
**29**	* 8-Epiloganic acid [4]	6.47	C_16_H_24_O_10_	375.12913	375.12970	−1.5	151.0753; 59.0124; 169.0860; 69.0331; 89.0229; 95.0488	I
**30**	* Salidroside	6.84	C_14_H_20_O_7_	299.11308	299.11365	−1.9	137.0232; 59.0124; 71.0124; 138.0549; 119.0489; 89.0229	PEG
**31**	* Glucosyringic acid	7.05	C_15_H_20_O_10_	359.09783	359.09836	−1.5	59.0124; 89.0229; 71.0124; 197.0445; 101.0229; 113.0230	PA
**32**	* Swertiamacroside	7.12	C_21_H_28_O_13_	487.14517	487.14609	−1.9	179.0341; 135.0439; 161.0234; 180.0375; 113.0231; 174.9553	PA
**33**	* Gentisoyl glucoside	7.18	C_14_H_20_O_8_	315.07161	315.07139	0.7	109.0281; 153.0182; 110.0315; 135.0075; 65.0383; 154.0216	PA
**34**	* Salicylic acid glucoside	7.51	C_13_H_16_O_8_	299.07670	299.07636	1.1	93.0332; 137.0232; 94.0364; 138.0266; 71.0124; 85.0281	PA
**35**	* Sucrose 6-benzoate	7.63	C_19_H_26_O_12_	445.13461	445.13531	−1.6	121.0282; 89.0229; 122.0315; 101.0229; 71.0124; 59.0125	PA
**36**	4′-*O*-Methylisoscutellarein 7-*O*-[6′-*O*-acetyl]-allosyl-(1→2)-[6′-*O*-acetyl]-glucoside	8.41	C_32_H_36_O_18_	707.18235	707.18274	−0.6	191.0552; 353.0877; 192.0586; 354.0911; 161.0231; 179.0339	F
**37**	Chlorogenic acid [18,20,25]	8.42	C_16_H_18_O_9_	353.08726	353.08783	−1.6	191.0552; 192.0586; 85.0280; 93.0332; 161.0235; 127.0388	PA
**38**	* Barlerin	8.76	C_19_H_28_O_12_	447.15026	447.15106	−1.8	161.0446; 269.1031; 101.0230; 71.0124; 113.0231; 89.0229	I
**39**	* Stachysoside E/G	9.28	C_30_H_38_O_17_	669.20308	669.20361	−0.8	163.0389; 187.0391; 325.0928; 205.0499; 145.0283; 181.0497	I
**40**	Unknown	9.34	C_18_H_28_O_12_	435.15026	435.15088	−1.4	59.0124; 167.0702; 346.3243; 108.5539; 221.9166; 116.5048	-
**41**	Apigenin 7-*O*-allosyl(1→2)-glucoside [16]	9.41	C_27_H_30_O_15_	593.15065	593.15137	−1.2	593.1515; 473.1089; 594.1549; 353.0667; 383.0772; 503.1198	F
**42**	Caffeic acid	9.44	C_9_H_8_O_4_	179.03444	179.03415	1.6	135.0441; 134.0361; 89.0383; 136.0473; 107.0490; 117.0333	PA
**43**	Ajugoside [3,18]	9.69	C_17_H_26_O_10_	389.14478	389.14542	−1.6	59.0124; 89.0231; 112.9843; 68.9943; 101.0229; 71.0124	I
**44**	7-*O*-acetyl-8-epiloganic acid [18]	9.72	C_18_H_26_O_11_	417.13969	417.14059	−2.2	59.0124; 89.0229; 107.0489; 193.0862; 71.0124; 151.0753	I
**45**	β-Hydroxyverbascoside [17,18]	10.25	C_29_H_36_O_16_	639.19252	639.19348	−1.5	161.0234; 179.0341; 621.1828; 639.1941; 622.1855; 459.1513	PEG
**46**	* Coumaroylmelittoside derivative	10.91	C_32_H_40_O_18_	711.21365	711.21417	−0.7	163.0390; 367.103; 187.0392; 205.0500; 145.0283; 181.0496	I
**47**	* N1, N10-Bis(*p*-coumaroyl)spermidine	11.28	C_25_H_31_N_3_O_4_	436.22363	436.22391	−0.6	119.0488; 316.1663; 145.0283; 290.1873; 317.1695; 120.0521	PA
**48**	Echinacoside/phlinoside A [23,25]	11.48	C_35_H_46_O_20_	785.25043	785.25116	−0.9	193.0499; 767.2409; 785.2502; 768.2443; 786.2529; 639.1931	PEG
**49**	Hypolaetin 7-*O*-allosyl(1→2)glucoside [18]	11.60	C_27_H_30_O_17_	625.14048	625.14099	−0.8	625.1407; 301.0352; 626.1445; 463.0881; 300.0272; 445.0777	F
**50**	* Dihydrodehydrodiconiferyl alcohol hexoside	11.66	C_26_H_34_O_11_	521.20229	521.20282	−1.0	329.1396; 330.1429; 175.0753; 177.0546; 71.0124; 193.0864	BF
**51**	Forsythoside B/Samioside/Lavandulifolioside [18,23,25]	11.76	C_34_H_44_O_19_	755.23986	755.24044	−0.8	755.2405; 756.2438; 757.2453; 593.2094; 161.0233; 594.2143	PEG
**52**	Verbascoside/isoverbascoside [2,18,23,25]	12.09	C_29_H_36_O_15_	623.19760	623.19812	−0.8	161.0233; 623.1984; 461.1664; 624.2015; 162.0269; 462.1700	PEG
**53**	* Vicenin-2 (6.8-diglucosylapigenin)	12.37	C_27_H_30_O_15_	593.15065	593.15155	−1.5	269.0457; 593.1517; 270.0490; 594.1548; 431.0984; 432.1029	F
**54**	* Quercetin-3*β*-D-glucoside [4]	12.41	C_21_H_20_O_12_	463.08766	463.08841	−1.6	300.0275; 301.0342; 161.0233; 302.0385; 463.0851; 151.0025	F
**55**	Verbascoside/isoverbascoside [2,18,23,25]	12.64	C_29_H_36_O_15_	623.19760	623.19812	−0.8	161.0233; 623.1982; 461.1668; 624.2015; 162.0268; 462.1712	PEG
**56**	Isoscutellarein 7-*O*-allosyl(1→2)-glucoside (All-Glc-ISC) [18,20]	12.79	C_27_H_30_O_16_	609.14557	609.14581	−0.4	285.0402; 609.1458; 286.0437; 429.0825; 610.1489; 284.0322	F
**57**	Isoscutellarein acetyl dissacharide [18]	12.84	C_29_H_32_O_17_	651.15613	651.15674	−0.9	651.1567; 652.1603; 609.1461; 285.0401; 610.1490; 286.0435	F
**58**	Glycosidic derivative of a methylether of acteoside [27]	12.89	C_36_H_48_O_20_	799.26608	799.26605	0.0	799.2664; 800.2699; 623.2181; 193.0496; 637.2153; 624.2218	PEG
**59**	Allysonoside [18,25]	13.07	C_35_H_46_O_19_	769.25551	769.25604	−0.7	769.2560; 770.2590; 638.2175; 593.2093; 637.2131; 193.0498	PEG
**60**	3′-*O*-Methylhypolaetin 7-*O*-allosyl(1→2)-glucoside [18,28]	13.13	C_28_H_32_O_17_	639.15613	639.15674	−1.0	315.0510; 639.1558; 316.054; 640.1589; 459.0933; 477.1038	F
**61**	Hypolaetin 7-*O*-[6‴-*O*-acetyl]-allosyl(1→2)-glucoside [18,20,25]	13.16	C_29_H_32_O_18_	667.15105	667.15155	−0.8	667.1515; 301.0351; 668.1549; 463.0880; 625.1407; 300.0274	F
**62**	* Asystoside	13.17	C_25_H_44_O_15_	583.26020	583.25946	1.3	289.1656; 161.0445; 451.2187; 101.0229; 421.2079; 71.0124	AAG
**63**	* Isoscutellarein 7-*O*-glucoside [3]	13.28	C_21_H_20_O_11_	447.09274	447.09323	−1.1	285.0402; 286.0437; 284.0326; 112.9843; 447.0921; 241.0497	F
**64**	Leucoseptoside A [18,23,25]	13.49	C_30_H_38_O_15_	637.21325	637.21381	−0.9	175.0391; 461.1666; 637.2139; 161.0234; 638.2178; 193.0498	PEG
**65**	* 2-(4-Hydroxyphenyl)-ethyl-(6-*O*-caffeoyl)-*β*-D-glucopyranoside	13.63	C_23_H_26_O_10_	461.14478	461.14572	−2.1	161.0233; 461.1455; 162.0267; 462.1493; 179.0341; 135.0439	PEG
**66**	* Methylhypolaetin glucoside	13.70	C_22_H_22_O_12_	477.10331	477.10394	−1.3	315.0509; 300.027; 316.0542; 301.0307; 314.0431; 477.1003	F
**67**	Apigenin 7-*O*-beta-D-glucoside [23,25]	13.77	C_21_H_20_O_10_	431.09783	431.09845	−1.4	268.0376; 269.044; 431.0981; 432.1019; 270.0490; 311.0545	F
**68**	Apigenin 7-*O*-[6‴-*O*-acetyl]-allosyl(1→2)-glucoside [25]	14.12	C_29_H_32_O_16_	635.16122	635.16150	−0.4	269.0454; 635.1616; 270.0488; 636.1651; 593.1498; 637.1656	F
**69**	Isoscutellarein 7-*O*-[6‴-*O*-acetyl]-allosyl(1→2)-glucoside [2,18,20]	14.39	C_29_H_32_O_17_	651.15613	651.15662	−0.7	285.0402; 651.1563; 429.0825; 286.0437; 652.1592; 284.0326	F
**70**	* 3′-*O*-Methylisoscutellarein 7-*O*-[6‴-*O*-acetyl]-allosyl(1→2)glucoside	14.52	C_30_H_34_O_17_	665.17178	665.17224	−0.7	299.0560; 623.1616; 624.1649; 300.0594; 665.1718; 666.1748	F
**71**	* Stachysoside D/Leonoside B	14.70	C_36_H_48_O_19_	783.27116	783.27142	−0.3	783.2717; 784.2749; 175.0391; 193.0498; 607.2245; 652.2327	PEG
**72**	4′-*O*-Methylhypolaetin 7-*O*-[6‴-*O*-acetyl]-allosyl(1→2)-glucoside [2,20]	14.76	C_30_H_34_O_18_	681.16670	681.16711	−0.6	315.0511; 681.1669; 316.0543; 682.1705; 639.1565; 459.0942	F
**73**	* 4′-*O*-Methyl-(−)-epigallocatechin 7-*O*-glucuronide	14.84	C_22_H_24_O_13_	495.11387	495.11456	−1.4	197.0448; 153.0546; 198.0481; 182.0211; 297.0614; 121.0282	F
**74**	* 1-Octen-3-yl primeveroside	14.92	C_19_H_34_O_10_	421.20738	421.20795	−1.4	71.0124; 101.0229; 113.0230; 85.0280; 73.0280; 161.0444	AAG
**75**	Martynoside [4,19,25,27]	15.29	C_31_H_40_O_15_	651.22890	651.22961	−1.1	175.0391; 651.2321; 193.0499; 176.0425; 475.1823; 652.2353	PEG
**76**	4′-*O*-Methylisoscutellarein 7-*O*-allosyl(1→2)glucoside [25]	15.48	C_28_H_32_O_16_	623.16122	623.16193	−1.1	299.0561; 300.0595; 623.1603; 624.1642; 112.9842; 161.0235	F
**77**	3′-*O*-Methylhypolaetin 7-*O*-[6‴-*O*-acetyl]-allosyl(1→2)glucoside [18,28]	15.98	C_30_H_34_O_18_	681.16670	681.16766	−1.4	315.051; 681.1679; 316.055; 682.1724; 501.1043; 519.1148	F
**78**	Hypolaetin 7-*O*-[2‴,6‴-di-*O*-acetyl]-allosyl(1→2)glucoside [18,28]	16.32	C_31_H_34_O_19_	709.16161	709.16180	−0.3	709.1619; 710.1654; 301.0351; 667.1522; 505.0992; 649.1404	F
**79**	* Tremasperin	17.22	C_30_H_34_O_16_	649.17687	649.17664	0.4	283.0611; 284.0644; 607.1667; 299.0561; 112.9841; 268.0363	F
**80**	4′-*O*-Methylisoscutellarein 7-*O*-[6‴-*O*-acetyl]-allosyl(1→2)glucoside [18,20,25]	17.37	C_30_H_34_O_17_	665.17178	665.17242	−1.0	299.0561; 665.1722; 300.0594; 666.1760; 101.0230; 461.1095	F
**81**	* Scutellarein/Isoscutellarein	17.49	C_15_H_10_O_6_	285.03992	285.04037	−1.6	133.0283; 285.0404; 151.0026; 175.0391; 107.0125; 149.0232	F
**82**	Isoscutellarein 7-*O*-[6‴-*O*-acetyl]-allosyl(1→2)-[6″-*O*-acetyl]-glucoside [18,25,28]	17.57	C_31_H_34_O_18_	693.16670	693.16705	−0.5	285.0404; 693.168; 471.0933; 633.1464; 651.1561; 284.0326	F
**83**	4′-*O*-Methylhypolaetin 7-*O*-[6‴-*O*-acetyl]-allosyl-(1→2)[6″-*O*-acetyl]-glucoside [2,18,29]	17.88	C_32_H_36_O_19_	723.17726	723.17773	−0.7	315.0511; 723.1779; 316.0543; 681.1673; 724.1811; 501.1039	F
**84**	* Proanthocyanidin dimer	18.15	C_30_H_26_O_12_	577.13461	577.13519	−1.0	269.0455; 270.0489; 577.1358; 578.1385; 145.0282; 307.0821	F
**85**	* Trihydroxy octadecadienoic acid	18.88	C_18_H_32_O_5_	327.21715	327.21774	−1.8	211.1334; 171.1017; 85.0281; 229.1442; 97.0645; 183.1381	FA
**86**	Apigenin-7-*O*-(6″-*O*-4-coumaroyl)-beta-glucoside [16,23,24,25]	19.91	C_30_H_26_O_12_	577.13461	577.13519	−1.0	269.0455; 145.0284; 431.0982; 413.0878; 577.1352; 270.0489	F
**87**	* Trihydroxy-octadecenoic acid	20.08	C_18_H_34_O_5_	329.23280	329.23331	−1.5	211.1334; 229.1441; 183.1382; 99.0801; 171.1018; 212.1367	FA
**88**	4′-*O*-Methylisoscutellarein 7-*O*-[6‴-*O*-acetyl]-allosyl(1→2)-[6″-*O*-acetyl]-glucoside [20,23,25,28,29]	20.55	C_32_H_36_O_18_	707.18235	707.18292	−0.8	299.0560; 707.1829; 300.0593; 708.1871; 101.0229; 665.1715	F
**89**	* Rosmanol	21.38	C_20_H_26_O_5_	345.17020	345.17005	0.4	301.1810; 283.1704; 302.1844; 284.1738; 61.9870; 258.1257	DT
**90**	* Cirsimaritin [4]	21.45	C_17_H_14_O_6_	313.07122	313.07163	−1.3	283.0247; 284.0280; 297.0403; 255.0297; 298.0466; 163.0026	F
**91**	* (−)-Usnicacid/Eupatorin	21.73	C_18_H_16_O_7_	343.08178	343.08215	−1.1	313.0355; 298.0118; 270.0169; 314.0388; 328.0588; 285.0404	F
**92**	* Genkwanin	21.84	C_16_H_12_O_5_	283.06065	283.06025	1.4	268.0376; 269.0409; 240.0420; 117.0331; 283.0614; 239.0344	F
**93**	* 4-Dodecylbenzenesulfonic acid	22.46	C_18_H_30_O_3_S	325.18374	325.18357	0.5	325.1845; 183.0113; 326.1877; 184.0181; 216.009; 197.0272	OSC
**94**	* Carnosol	22.48	C_20_H_26_O_4_	329.17529	329.17519	0.3	285.1860; 286.18933; 201.0914; 270.1627; 214.0999; 269.1543	DT
**95**	* Hydroxylinoleic acid	22.73	C_18_H_32_O_3_	295.22732	295.22781	−1.7	98.9544; 277.2171; 61.9869; 195.1384; 171.1016; 96.9587	FA
**96**	* Dodecyl sulfate	22.74	C_12_H_26_O_4_S	265.14736	265.14786	−1.9	96.9587; 265.1479; 79.9559; 97.9577; 95.9508; 266.1515	OSC
**97**	* Carnosic acid	22.94	C_20_H_28_O_4_	331.19094	331.19056	1.1	332.1867; 286.1809; 314.1765; 287.2008; 96.9584; 331.2667	DT
**98**	* Lauryl ether sulphate	23.98	C_14_H_30_O_5_S	309.17357	309.17401	−1.4	96.9586; 309.1739; 79.9558; 310.1771; 122.974; 94.9794	OSC
**99**	* Bis(2-ethylhexyl) adipate	24.35	C_22_H_42_O_4_	369.30049	369.30075	−0.7	72.9916; 75.0073; 369.3006; 59.0124; 293.2846; 323.2971	CA
**100**	* Myristyl sulfate	24.36	C_14_H_30_O_4_S	293.17866	293.17902	−1.2	96.9587; 293.1791; 221.1539; 220.1462; 294.1826; 79.9558	OSC
**101**	* Diisononyl adipate	24.37	C_24_H_46_O_4_	397.33179	397.33212	−0.9	59.0124; 397.226; 351.3631; 96.9587; 72.9916; 397.3729	CA
**102**	* 16-Hydroxyhexadecanoic acid	24.51	C_16_H_32_O_3_	271.22732	271.22784	−1.9	225.2218; 223.2063; 226.2252; 197.1903; 221.1908; 224.2096	FA

Classes: AA, amino acids; AAG, aliphatic alcohol glycosides; BF, benzofurans; CA, carboxylic acids; DT, diterpenoids; F, flavonoids and derivatives; FA, fatty acids; I, iridoids; N, nucleosides; OSC, organosulfur compounds; PA, phenolic acids and derivatives; PEG, phenylethanoid glycosides; S, sugar acids and saccharides. Tentative identification based on MS, MS^2^ and literature data for *Sideritis* species or *Lamiaceae* family, and if not available, Pubchem or HMDB databases. * symbol before the compound name indicates that the compound has been identified in studied *Sideritis* species for the first time.

## Data Availability

Data is contained within the article.

## Sample Availability

Samples of the compounds are not available from the authors.
